# Mental fatigue impairs time trial performance in sub-elite under 23 cyclists

**DOI:** 10.1371/journal.pone.0218405

**Published:** 2019-06-17

**Authors:** Luca Filipas, Gabriele Gallo, Luca Pollastri, Antonio La Torre

**Affiliations:** 1 Department of Biomedical Sciences for Health, Università degli Studi di Milano, Milan, Italy; 2 School of Sport Science, Università degli Studi di Milano, Milan, Italy; 3 PENTAVIS, Laboratory of Sport Sciences, Lecco, Italy; 4 IRCCS Istituto Ortopedico Galeazzi, Milan, Italy; University of Bourgogne France Comté, FRANCE

## Abstract

**Purpose:**

This study investigates the effect of a mentally demanding response inhibitory task on time trial performance in sub-elite under 23 cyclists.

**Methods:**

Ten under 23 road cyclists completed two separate testing sessions during which they performed two different cognitive tasks before completing a 30-min time trial on the cycle ergometer. In the experimental condition, 30 min of a standard cognitive task (Stroop task) was used to elicit mental fatigue; in the control condition, a non-demanding activity was carried out. Subjective workload and mood were measured before and after the treatments, and motivation was recorded before the time-trial. During the time trial, power, cadence, heart rate, and rate of perceived exertion were assessed. Blood lactate concentrations and heart rate variability (using the root mean square of the successive differences) were measured before and after the time trial.

**Results:**

The Stroop task was rated more mentally (P < 0.001) and temporally (P < 0.001) demanding, effortful (P < 0.001), and frustrating (P = 0.001) than the control task; fatigue (P = 0.002) and vigor (P = 0.018) after the cognitive tasks were respectively higher and lower than in the control task. Mean power output (P = 0.007) and cadence (P = 0.043) were negatively affected by the Stroop task, while heart rate (P = 0.349), rating of perceived exertion (P = 0.710), blood lactate concentration (P = 0.850), and root mean square of the successive differences (P = 0.355) did not differ between the two conditions.

**Conclusion:**

A mentally demanding activity reduced the subsequent physical performance in sub-elite under 23 cyclists. Thus, avoiding cognitive efforts before training and races could improve performance of high-level athletes.

## Introduction

Mental fatigue is defined as a psychobiological state that may arise during or after prolonged cognitive activities, and it is characterized by the feelings of tiredness, decreased commitment, and increased aversion to continue the current activity [1]. Acute mental fatigue has a negative effect on submaximal endurance performance [2]: several studies in this area showed that mental fatigue impairs whole body and muscular physical endurance performance, both in close-ended tasks [3,4] and in open-ended tasks [5–7]. The underlying mechanism that can explain why mental fatigue impairs endurance performance seems to be an increased perception of effort for external and internal loads. For further explanation on this topic, see the review of Van Cutsem et al. [2].

The physiological reasons for an increased rating of perceived exertion (RPE)-load ratio are not yet fully understood. A model has recently been proposed, based on the interaction between the adenosine neuromodulator and the encephalic A1 receptors [8].

Beyond the definition of the physiological mechanisms of mental fatigue, it is important to understand whether the ability to resist mental fatigue is associated with a certain degree of trainability. In a recent study, Martin and colleagues [6] demonstrated that mental fatigue impairs endurance performance (measured as power output during a 20-min time trial) in recreational cyclists, but not in professional cyclists. The authors hypothesized that superior resistance to mental fatigue may be one of the characteristics that distinguishes successful endurance athletes, together with traditional physiological and psychological abilities. It is difficult to predict the nature of this greater fatigue, resistance and to understand if this peculiar ability could be trainable. The improved performance of elite cyclists may be a result of training as there are a host of beneficial changes that occur in the brain as a result of aerobic training [8–12].

Although the physiological mechanisms of mental fatigue need to be further investigated, a few studies reported that acute mental fatigue can also influence the autonomic regulation of heart rate (HR). Mental fatigue induces a sympathetic hyperactivity, a decrease in parasympathetic activity and, therefore, a reduction in heart rate variability (HRV) [13,14]. During the last 25 years, HRV has been widely used as a non-invasive method to estimate cardiac autonomic regulation, which may reflect the activity of the autonomic nervous system [15]. In sports, rest or post exercise HRV is growing in importance as an objective and rational method for the quantification of training load in endurance athletes [16,17]. Despite two studies demonstrated acute changes in HRV during mentally fatiguing protocols [13,14], a recent study in young swimmers showed that post treatment and post exercise HRV values could be unchanged after a mental effort [18], highlighting potential issues in the quantification of training load in mentally fatigued athletes. Athletes experience mental fatigue conditions before training or competitions (i.e. use of smartphone, long tactical sessions), therefore understand the impact of this condition on training load could be useful to evaluate it correctly.

To date, no studies have investigated the effect of mental demanding tasks on sub-elite endurance athletes, a category that could have similarities and differences to both elite and recreational athletes. Therefore, the first aim of the present study was to further investigate the effect of a mental demanding and response inhibitory task on time trial performance in sub-elite under 23 cyclists. Based on previous findings of an association between performance level and inhibitory control in elite cyclists [6] and ultramarathon runners [19], we hypothesized that under 23 cyclists’ performance would be negatively affected by the mentally demanding task. The second aim of this study was to identify possible alterations in the autonomic control of HR following a prolonged mentally demanding task. The influence of mental fatigue on HRV could be crucial to evaluate the efficacy of HRV as a predictor of training load in mentally fatigued athletes. We hypothesized that mental fatigue would not alter HRV values, as reported in the research on young swimmer, the only study that investigated HRV and mental fatigue in a sport context [18].

## Materials and methods

### Subjects

Ten under 23, male road cyclists (20.0 ± 1.2 yr, 66.1 ± 7.6 kg, 180.4 ± 5.6 cm, VO_2max_ 69.0 ± 4.4 mL · min^–1^ · kg^–1^, peak power output 380 ± 39 W, > 4 training sessions per week, > 300 km per week, > 3 years of cycling experience) voluntarily participated in this study. Participants were members of different under 23 cycling teams affiliated to the Italian Cycling Federation. Considering each participant’s VO_2max_ and training history, and in line with guidelines designed to help describe the performance level of participants in sports science research [20], the subjects were classified as performance level 4 (well-trained). Eligibility criteria were as follows: free from any known medical diseases, injuries, color vision deficiencies and learning disorders, free from any medication. The study design and procedures were approved by the Università degli Studi di Milano Ethics Committee and followed the ethical principles for medical research involving human subjects set by the World Medical Association Declaration of Helsinki. After ethical approval, written informed consent and medical declaration were obtained from the participants in line with the procedures set by the local Institution’s Research Ethics Committee. Subjects were informed of the procedures and potential risks involved. They were also informed that they were free to withdraw from the study at any time.

### Experimental design

A randomised counterbalanced cross-over design was used for the experimental component of the present study. The order of the experimental treatments (intervention; control) was randomly allocated based on balanced permutations generated by a web-based computer program (www.randomization.com).

### Experimental overview

**Fig 1:**
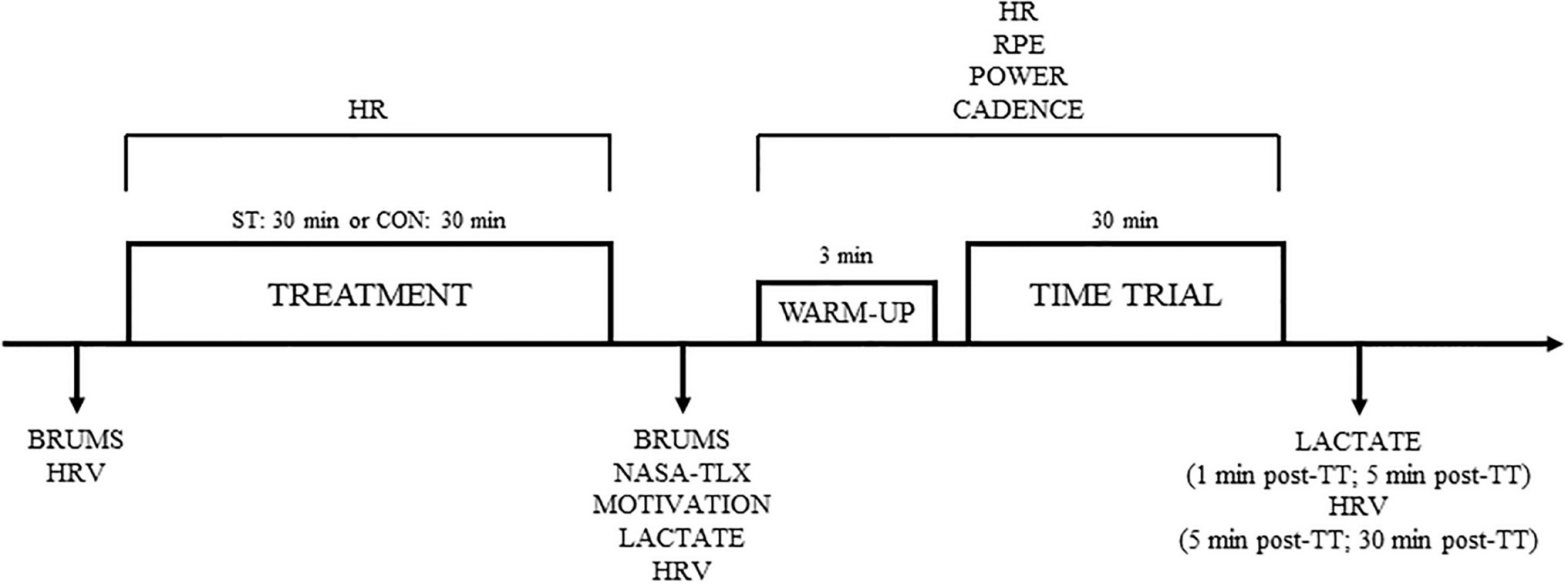
Schematic of the visits 3 and 4. HR–Heart Rate; HRV–Heart Rate Variability; RPE–Rating of Perceived Exertion; ST–Stroop Task; CON–Control.

Subjects performed four testing sessions on four different occasions, in a period no longer than three weeks between the first and last visit. Visits were carried out at the university laboratory. Cognitive and physical tasks were performed in an isolated and air-conditioned room, at the constant temperature of 19 ± 1 °C and at a relative humidity of about 40–50%. Prior to each visit, participants were instructed to sleep for at least 8 h, refrain from the consumption of alcohol and caffeine, and avoid any vigorous exercise for the 36-h preceding the testing sessions. Participants were also instructed to avoid any mentally demanding tasks the day of the testing sessions. Each participant carried out the visits individually and at the same time of day (within 1 h period, between 9:00 and 12:00). During visit 1, participants weight and height were measured. Afterwards, they familiarized with the procedures employed for the experimental sessions, i.e. the Stroop task (for the time needed to reach a minimum of 95% of accuracy), psychological questionnaires, and the physical task, i.e. a time trial on a cycling ergometer. During visit 2, participants completed an incremental exercise test to determine their VO_2max_. A graphical representation of visit 3 and 4 is shown in Fig 1.

### Experimental treatment

The mental fatigue condition consisted in a 30-min modified Stroop color-word task. The Stroop task demands response inhibition and sustained attention [21] and has previously been shown to induce mental fatigue [2]. The modified Stroop task used in our protocol was the same version and had the same implementing rules described by Martin et al. [6]. Participants were instructed to respond as quickly and as accurately as possible. Participants familiarized with the Stroop task for 5 min during the preliminary visit. Additionally, 24 practice attempts prior to the experimental task were allowed, to ensure the participants fully understood the instructions. The reaction time of the correct responses and accuracy were averaged for 6 blocks of 5 minutes.

The control trial involved watching a 30-min video regarding amphibious excavators under the same conditions as the Stroop task.

### Physical tests

During the second visit, participants underwent an incremental exercise test on a cycle ergometer (Cyclus 2, RBM elektronik automation GmbH, Leipzig) to assess their VO_2max_. The incremental exercise test began at 100 W and increased by 25 W every 30 s until volitional exhaustion.

Participants performed the time trial during the following two visits to the laboratory. A standardized warm-up at the constant power output of 100 W was completed by all participants prior to each time trial. Both were performed on the Cyclus 2 ergometer. Participants were instructed to cover as much distance as possible over 30 min. The time trial began in a standard gear; however, participants were free to alter gearing throughout the time trial. A timer was placed to the front right of participants and remained visible during the time trial; participants were blinded to all other performance and physiological data. After the test, mean power output and mean cadence were analyzed in 10 blocks of 3 min each. Average power and cadence during the whole-time trial were also recorded.

### Physiological measures

Capillary blood samples were collected immediately before the warm-up and two times after completion of time trial (1 min and 5 min) during visits 3 and 4. Samples were analyzed immediately for blood lactate concentration using the Lactate Pro 2 (Arkray, Japan) analyzer. During visits 3 and 4, HR was recorded during the final 10 s of the 10 blocks of 3 min each using a HR monitor fitted with a chest strap.

### Psychological measures

RPE was registered during the final 10 s of the 10 blocks of 3 min each with the 11-point CR10 developed by Borg [22]. Participants were familiar with the scale as it had been employed during their daily training sessions for at least six months prior to the tests.

The Brunel Mood Scale (BRUMS) developed by Terry et al. [23] was used to assess changes in start and post-treatment mood. The questionnaire consists of 24 items divided into 6 subscales related to mood (Depression, Fatigue, Vigor, Confusion, Anger, Tension). Participants were asked to rate each item on a 5-point Likert scale (from 0 = not at all, to 4 = extremely) according to their current mood (“How do you feel right now?”). Each subscale score, with four relevant items, could range from 0 to 16.

The subjective workload was recorded after the treatments with the Italian version of the National Aeronautics and Space Administration Training Load Index (NASA-TLX) [24]. It involves a multi-dimensional rating procedure with 6 subscales (Mental demand, Physical demand, Temporal demand, Effort, Frustration). Subjects were asked to rate each of them on a 0 to 20 scale anchored by bipolar descriptors (high/low). Each score was multiplied by 5 so that the final score of each subscale would range from 0 to 100.

Motivation toward time trials was measured after the treatments with a single item on a 5-point Likert scale (0 = not at all, 1 = a little bit, 2 = somewhat, 3 = very much, 4 = extremely) [6].

### HRV

HRV was measured at baseline, after the Stroop task and twice after the time trial (5 min and 30 min) in both conditions. For all measurements, the participants remained in a lying position for five minutes, with a normal breathing rate, in silence and with no body movements. To collect the HR data, a Polar T61 chest belt connected to a recording watch (Polar Electro, Kempele, Finland) recorded a beat-to-beat HR at a 1-ms time resolution [25]. Occasional ectopic beats were visually identified and manually replaced with interpolated adjacent R-R interval values. To identify the HRV in the time-domain, average R-R intervals (RR mean) and the root mean square of successive differences between adjacent R-R intervals (RMSSD) were analyzed. All analyses were performed with Kubios HRV Analysis Software v2.2 (Biosignal analysis and medical imaging group, University of Eastern Finland, Finland) [26].

### Statistical analysis

All data are presented as mean ± standard deviation. Assumptions of statistical tests such as normal distribution and sphericity of data were checked as appropriate. Greenhouse-Geisser correction to the degrees of freedom was applied when violation to sphericity was present. One-way ANOVA was used to determine the effects of time for reaction time and accuracy during the Stroop task. Paired sample t tests were used to determine the effects of condition on the mean HR during the Stroop task, NASA TLX subscales, motivation related to the time trial, and average power, cadence and HR during the time trial. Repeated measures ANOVAs were used to determine the effects of condition and time for blood lactate concentration, RMSSD, mood subscales, and HR, RPE, cadence and power output during the time trial. Bonferroni tests were used if significant interactions were found. Additionally, the effect size for each statistical test is reported as partial eta squared (η^2^p), using the small = 0.02, medium = 0.13 and large = 0.26 interpretation for effect size [27]. Significance was set at 0.05 (2-tailed). All data analysis was conducted using the statistical packages for social science (SPSS version 24).

## Results

### Stroop task performance

There were no significant main effects of time on accuracy (overall grand mean: 95.3 ± 3.1%, *P* = 0.733, η^2^p = 0.058) and reaction time (overall grand mean: 1141 ± 206 ms, *P* = 0.362, η^2^p = 0.100). There was no significant difference also in the mean HR during Stroop task and control condition (overall mean Stroop task: 49 ± 10 bpm, overall mean control: 50 ± 9 bpm, *P* = 0.760).

### Psychological responses

**Fig 2:**
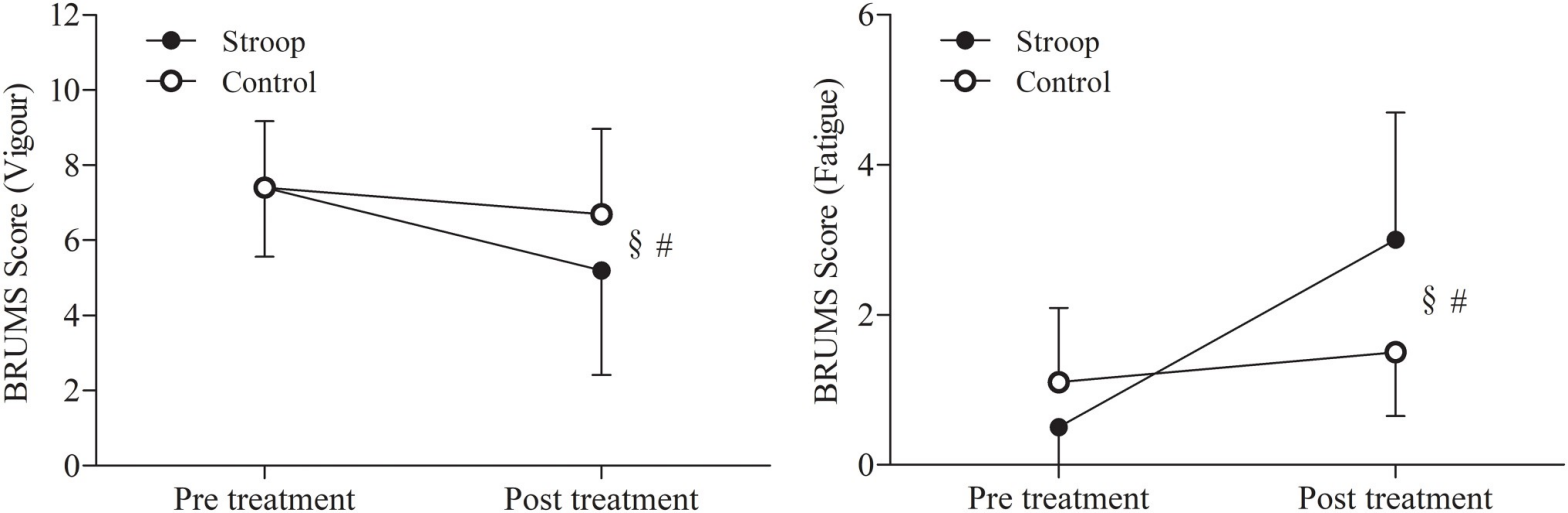
Effect of prior cognitive task on BRUMS vigor (left) and fatigue (right) index. Significant interactions condition x time (§, *P* < 0.05) and main effects of time (#, *P* < 0.05). Data are presented as mean ± SD.

The vigor and fatigue scale of the BRUMS revealed a significant condition x time interaction (vigor: *P* = 0.018, η^2^p = 0.479, fatigue: *P* = 0.002, η^2^p = 0.678). The follow-up tests revealed that the vigor scale decreased, and fatigue scale increased over time in both conditions. (vigor: P = 0.005, η^2^p = 0.605, fatigue: P = 0.001, η^2^p = 0.744). However, the Stroop task condition showed a greater decrease in vigor scale and increase in fatigue scale compared to the control (Fig 2).

NASA-TLX showed a significantly higher mental demand (*P* < 0.001), temporal demand (*P* < 0.001), effort (*P* < 0.001) and frustration (*P* = 0.001) after the Stroop task compared to the control task. Physical demand and performance did not differ between the two conditions (respectively *P* = 0.560 and *P* = 1.000). Data of subjective workload are reported in Table 1.

**Table 1:** NASA-TLX subjective workload values for the five subscales between the two conditions. Data are presented as mean ± SD.

	NASA-TLX workload					
	Mental demand	Physical demand	Temporal demand	Performance	Effort	Frustration
Stroop task	68 ± 14	13 ± 10	71 ± 17	25 ± 13	77 ± 9	46 ± 12
Control	34 ± 19	11 ± 6	35 ± 18	25 ± 13	33 ± 16	25 ± 14
*P*	< 0.001	0.560	< 0.001	1.000	< 0.001	< 0.001

Motivation for the time trial did not differ significantly between conditions (*P* = 0.443). Values were 3.3 ± 0.9 and 3.5 ± 0.8 for the Stroop task and the control condition respectively.

### Time trial performance

**Fig 3:**
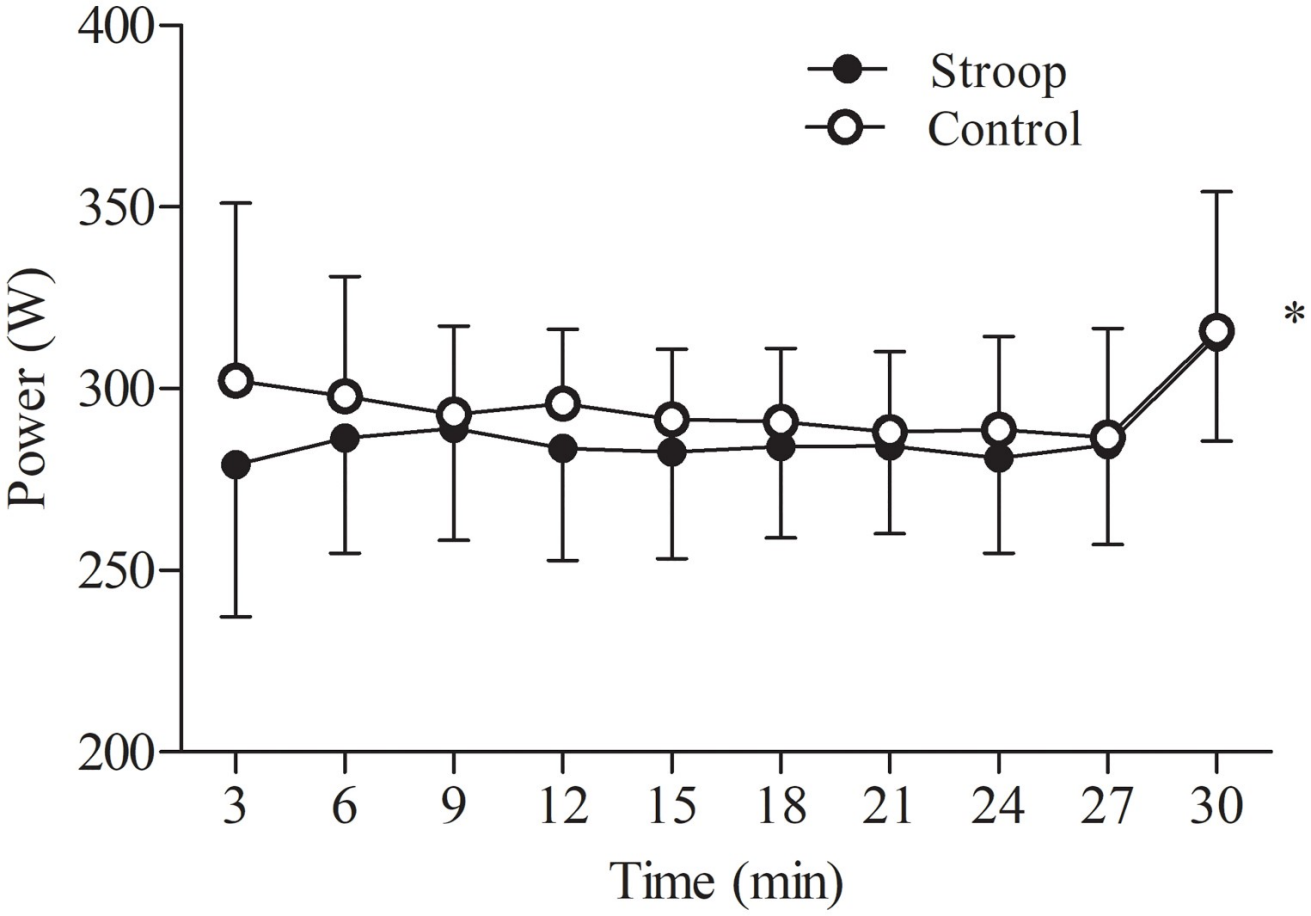
Effect of prior cognitive task on power output during the 30-min time trial. Significant main effect of condition (*, *P* < 0.05). Data are presented as mean ± SD.

There was no significant interaction condition x time (*P* = 0.234, η^2^p = 0.129) for power output during the time trial. However, there was a main effect of condition (Fig 3), with a lower power output recorded after the Stroop task compared to control condition (overall mean Stroop task: 287 ± 23 W, overall mean control: 295 ± 23 W, *P* = 0.007, η^2^p = 0.574). There was no significant main effect of time (*P* = 0.120, η^2^p = 0.207).

**Fig 4:**
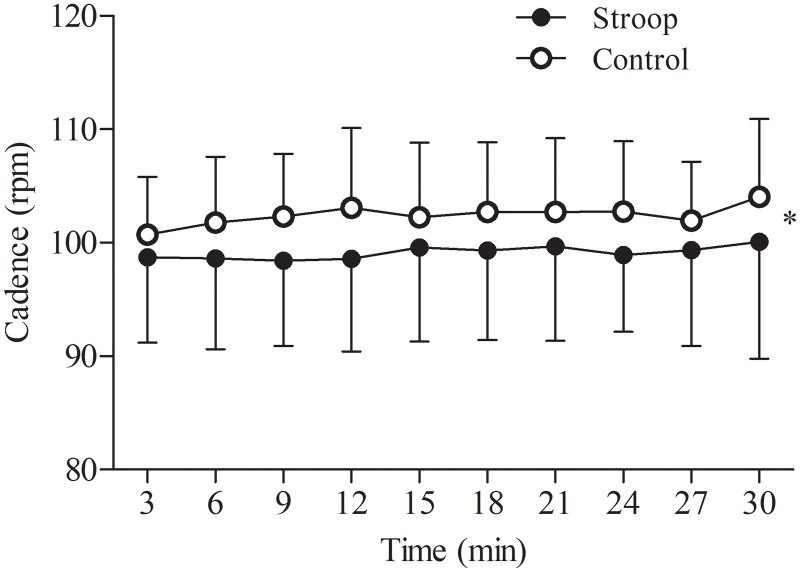
Effect of prior cognitive task on cadence during the 30-min time trial. Significant main effect of condition (*, *P* < 0.05). Data are presented as mean ± SD.

There was no significant interaction condition x time (*P* = 0.508, η^2^p = 0.081) for cadence during the time trial. However, there was a main effect of condition (Fig 4), with a significant lower cadence after the Stroop task compared to the control condition (overall mean Stroop task: 100 ± 7 rpm, overall mean control: 102 ± 6 rpm, *P* = 0.043, η^2^p = 0.382). There was no significant main effect of time (*P* = 0.373, η^2^p = 0.106).

### Physiological and perceptual responses during the time trial

There was no significant interaction condition x time (*P* = 0.919, η^2^p = 0.002) for blood lactate concentrations. There was no main effect of condition (*P* = 0.850, η^2^p = 0.004), but lactate was higher after time trial compared to baseline (*P* < 0.001, η^2^p = 0.848). Mean blood lactate concentrations for the Stroop task and the control condition were respectively 1.3 ± 0.2 mmol.l^-1^ and 1.3 ± 0.2 mmol.l^-1^ at the baseline, 7.7 ± 3.5 mmol.l^-1^ and 7.8 ± 2.6 mmol.l^-1^ one minute after the time trial, 6.7 ± 2.5 mmol.l^-1^ and 6.9 ± 2.6 mmol.l^-1^ five minutes after the time trial.

There was no significant condition x time interaction (*P* = 0.616, η^2^p = 0.054) for HR during the time trial. There was no main effect of condition (*P* = 0.349, η^2^p = 0.098). However, HR increased over time in both conditions (overall mean Stroop task: 172 ± 6 bpm, overall mean control: 173 ± 7 bpm, *P* < 0.001, η^2^p = 0.847).

**Fig 5:**
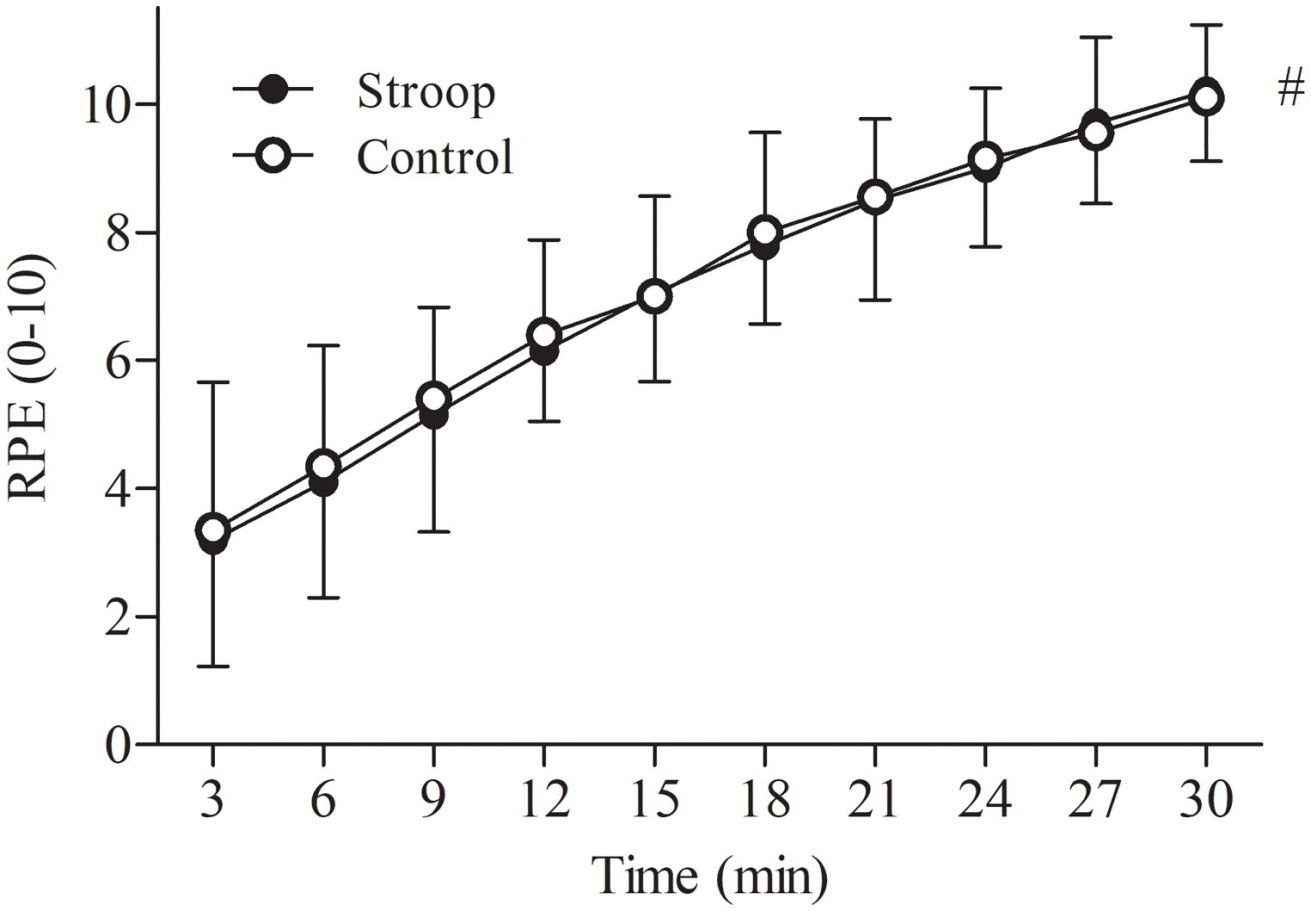
Effect of prior cognitive task on rating of perceived exertion (RPE) during the 30-min time trial. Significant main effect of time (#, *P* < 0.05). Data are presented as mean ± SD.

There was no significant condition x time interaction (*P* = 0.694, η^2^p = 0.054) for RPE during the time trial (Fig 5). There was no significant main effect of condition (*P* = 0.710, η^2^p = 0.016). However, RPE increased over time in both conditions (*P* < 0.001, η^2^p = 0.851).

### HRV responses

There was no significant condition x time interaction (*P* = 0.711, η^2^p = 0.049) for RMSSD values. No main effect of condition was found (*P* = 0.392, η^2^p = 0.082), but only a main effect of time (*P* < 0.001, η^2^p = 0.890). Specifically, HRV decreased more 5 min after the time trial, compared to 30 min. Mean RMSSD values for the Stroop task and the control condition were respectively 62.2 ± 14.2 ms and 63.1 ± 14.7 ms at baseline, 60.4 ± 17.7 ms and 62.8 ± 17.4 ms after the Stroop tasks, 6.5 ± 4.4 ms and 6.0 ± 2.5 ms five minutes after the time trial, 20.8 ± 17.0 ms and 22.4 ± 19.4 ms thirty minutes after the time trial.

## Discussion

The first aim of this study was to investigate the effect of a mentally demanding response inhibitory task on time trial performance in sub-elite under 23 cyclists. In accordance with our hypotheses, results suggest that the mentally demanding task impaired endurance performance via a reduction in average power output and cadence during the 30-min cycling time trial, without a change in physiological and perceptual responses. The second aim of this research was to identify possible alterations in the autonomic control of HR following a prolonged mental demanding task. As hypothesized, mental fatigue did not alter HRV values, both after the Stroop tasks and the time trial.

The two psychological questionnaires (BRUMS and NASA-TLX) highlighted that mental fatigue was induced by the Stroop task, even if accuracy and reaction time did not change significantly during the response inhibition task. A similar result was previously found in other research on the same topic [4,28]. The authors employed two different intervention tasks to induce mental fatigue. None of these affected accuracy and reaction time during the task. However, BRUMS [4] and NASA-TLX [28] showed an increased mental fatigue after the task compared to the control condition. The findings of these studies suggest that the subjects probably drained themselves trying to keep the same reaction time and accuracy during the Stroop tasks. Comparing the Stroop task performances with the study by Martin et al. [6], we found that reaction time in the under 23 cyclists tended to be constant throughout the task, as it did in the elite cyclists. Globally, the higher reaction time of our subjects could explain the lack of reduction in Stroop task performance (greater focus on overall accuracy). Therefore, the use of questionnaires, associated with the analysis of reaction time and accuracy, could probably help to check the effectiveness of the Stroop task to induce mental fatigue.

The response inhibition task significantly impaired performance during the 30-min time trial, in terms of power output and cadence. Although the time trails are slightly different in terms of duration (20 min vs 30 min), we found some similarities with recreational cyclists in Martin et al. [6]: sub-elite under 23 cyclists were negatively affected by a mentally demanding task. This confirms that this group of cyclists has some similarities to recreational cyclists, but also to professional ones. Indeed, the decrease of performance between control and mental fatigue condition in under 23 athletes is relatively small (~ 2.8%), but almost exactly in the middle between the ~ 0.9% reduction of elite cyclists and the ~ 5.8% reduction for the recreational cyclists. Acknowledging the limitation of this descriptive comparison, we could assume a progressive greater tolerance to mental fatigue from low to high level endurance athletes.

As found in previous studies [29], both HR and blood lactate concentrations remained similar in the mental fatigue and control conditions. Indeed, according to the literature on this topic [2], mental fatigue seems to be able to alter endurance performance without altering any exercise-induced physiological parameter. Interestingly, but not surprisingly, RPE was also unaffected by mental fatigue. The current general opinion is that endurance performance is impaired by mental fatigue and this is predominantly mediated by the higher than normal perceived exertion during exercise [2]. This is the situation of a time to task failure test, where power output remains constant through the test. In a time-trial test, power output fluctuates during the test and the pacing strategy could affect the perception of effort [30,31]. In the present study, the subjects perceived a similar effort at each time point in the two conditions, but power output was higher in the control condition than in the mental fatigue condition. Therefore, mental fatigue induced a higher RPE/power output ratio. This finding confirms that perceived exertion plays a key role in the reduction in endurance performance after a mental fatiguing task. The underlying mechanisms behind the increased perceived exertion induced by fatigue remain unclear, but recent studies [8,29] speculate that an increase in extracellular concentrations of adenosine caused by prior physical exertion could explain the increased perceived exertion caused by mental fatigue.

The lower cadence found after mental fatigue was unexpected because no other study found a similar result. Therefore, we can only speculate that a possible explanation could lie in the same mechanism of template RPE discussed above. Different studies reported that higher RPE is traditionally associated with higher cycling cadence [32,33]. Hence, the higher relative perception of fatigue after the Stroop task could have induced a reduction of cadence (accompanied by a reduction in power output) to restore an appropriate RPE trajectory.

The mental fatigue protocol did not change HRV in the present study. As such, changes in HRV cannot be attributed to any acute physical impairment caused by mental fatigue. This result confirms what was found by Penna and colleagues [18] in young swimmers, but contrasts with other two previous studies [13,14] that showed a reduced HRV during a mental fatiguing task. Probably, some changes would have been found if we had measured HRV during the Stroop task. But, our goal was to check if HRV could be a good method to predict training load in a mentally fatigued condition. Ultimately, HRV seems an unsensitive marker of mental fatigue in well-trained athletes.

### Practical applications

The findings of this study are important for coaches and professionals who are responsible for the planning and execution of training programs. Athletes should avoid cognitive efforts before training and races to prevent negative effects on their endurance performances. This is even more important for this this specific age group, because they suffer from mental fatigue daily at school or at work and could experience this condition more often then professional athletes. Moreover, the results of the present study highlight that HRV cannot be considered a useful measure to evaluate the additional load caused by mental fatigue.

## Conclusions

Previous studies showed that mental fatigue impairs physical performance in different endurance sports, especially in recreational and amateur athletes. This investigation highlights that a mentally demanding activity reduced the subsequent physical performance in sub-elite under 23 cyclists.

## Supporting information

S1 File Performance, physiological and psychological data. (XLSX)

S2 File Stroop performance data. (XLSX)
